# Pediatric Left Posteroseptal Accessory Pathway Ablation from Giant Coronary Sinus with Persistent Left Superior Cava

**DOI:** 10.3390/jcdd9040109

**Published:** 2022-04-06

**Authors:** José Cruzalegui, Sergi Cesar, Oscar Campuzano, Victoria Fiol, Josep Brugada, Georgia Sarquella-Brugada

**Affiliations:** 1 Pediatric Arrhythmias, Inherited Cardiac Diseases and Sudden Death Unit, Cardiology Department, Sant Joan de Déu Hospital de Barcelona, 08950 Barcelona, Spain; josecarlos.cruzalegui@sjd.es (J.C.); sergi.cesar@gmail.com (S.C.); jvfiolramis@gmail.com (V.F.); jbrugada@clinic.cat (J.B.); 2 European Reference Network for Rare, Low Prevalence and Complex Diseases of the Heart (ERN GUARD-Heart), 1105 AZ Amsterdam, The Netherlands; 3 Arrítmies Pediàtriques, Cardiologia Genètica i Mort Sobtada, Malalties Cardiovasculars en el Desenvolupament, Institut de Recerca Sant Joan de Déu, Esplugues de Llobregat, 08950 Barcelona, Spain; 4 Medical Science Department, School of Medicine, Universitat de Girona, 17003 Girona, Spain; oscar@brugada.org; 5 Cardiovascular Genetics Center, University of Girona-IDIBGI, 17190 Girona, Spain; 6 Centro de Investigación Biomédica en Red, Enfermedades Cardiovasculares (CIBERCV), 28029 Madrid, Spain; 7 Arrhythmia Section, Cardiology Department, Hospital Clínic, Universitat de Barcelona, 08036 Barcelona, Spain

**Keywords:** Wolff-Parkinson-White, pediatric accessory pathway, radiofrequency ablation, coronary sinus, persistent left superior vena cava

## Abstract

We report a pediatric patient with persistent left superior vena cava and a D-transposition of great arteries, which is an uncommon relation. It is crucial to know the anatomy of the persistent left superior vena cava and the dilated coronary sinus to plan the mapping techniques in cases of posterior accessory pathways.

## 1. Introduction

A persistent left superior vena cava (PLSVC) is a rare congenital heart anomaly present in nearly 0.21–0.6% of the general population and in 1.3–3.8% associated with other congenital heart defects [1,2]. In nearly 90% of cases, the PLSVC connects to the coronary sinus (CS), widening its structure and drains into the right atrium (RA). In the other 10% of cases, the PLSVC drains into the left atrium through a partially or completely unroofed coronary sinus, or even rarely through the left pulmonary veins or directly into the left atrial appendage [3].

In the most common form of PLSVC (PLSVC draining in the CS into the RA), the dilated CS distorts the anatomy of the heart and the intracardiac spatial relations making the correct mapping of left atrio-ventricular (AV) accessory pathways difficult [2]. The importance of a PLSVC and the dilated CS for ablation of a left AP is shown in this pediatric case report. 

## 2. Case Presentation

An 11-year-old girl with a personal history of prenatally diagnosed D-transposition of great arteries (TGA) in situs solitus, ventricular septal defect (VSD) and PLSVC underwent arterial switch and VSD closure at the age of 2-weeks-old. After the initial surgery, she needed three percutaneous pulmonary angioplasties (at 1, 4 and 5 years of age) and a surgical angioplasty of the pulmonary artery (at 5 years of age) because of pulmonary supravalvular stenosis. The last echocardiography showed a moderate tricuspid valve regurgitation, mild pulmonary (valvular and supravalvular) stenosis and a dilated coronary sinus with no atrial septal defect.

**Figure 1:**
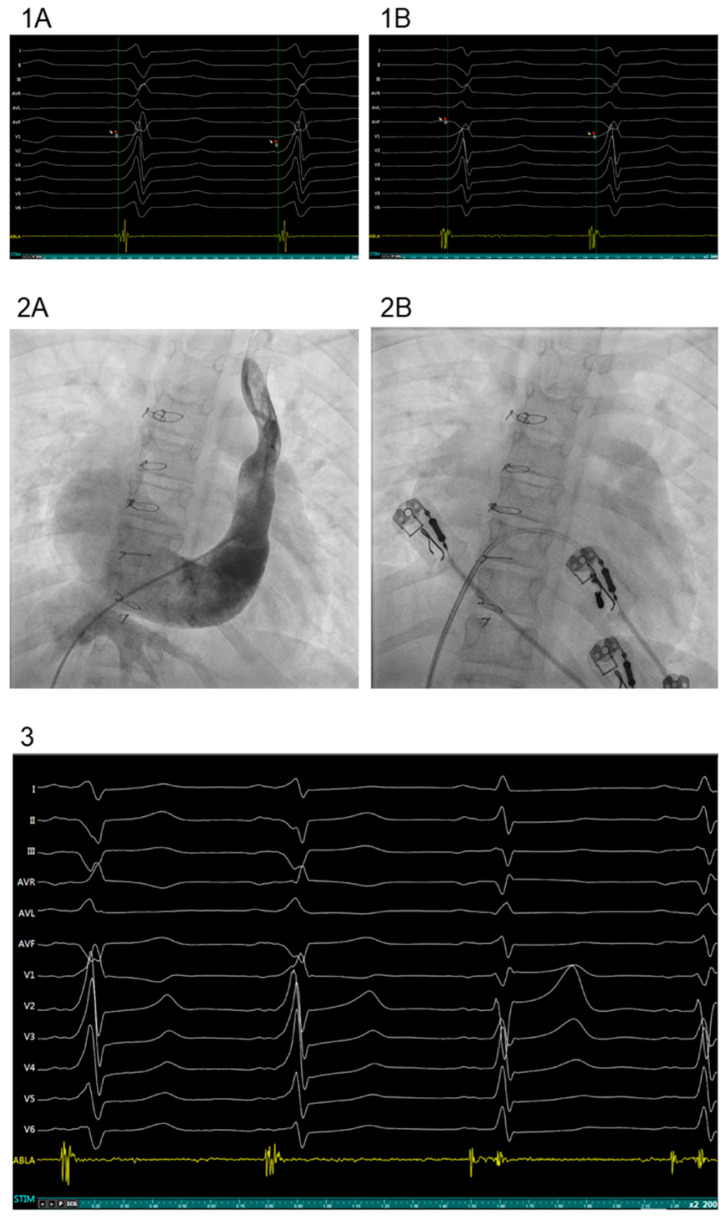
(**1A**) During EPS, delta wave and QRS are (+) in lead V1, and (−) in inferior leads suggesting a left posteroseptal accessory pathway. Mapping the MA from the left side (retroaortic), showed earliest ventricle activation at the beginning of the delta wave. (**1B**) Mapping the MA from inside the coronary sinus showed the earliest ventricle activation before the delta wave. (**2A**) Venous angiography at the persistent left superior vena cava showed contrast flowing to the right atrium through a dilated coronary sinus. (**2B**) Ablation catheter located at the point of effective ablation deep inside the dilated coronary sinus. (**3**) After mapping inside the giant CS, RF application during sinus rhythm at the earliest ventricular activation point removed the accessory pathway potential, showing separation of the atrial and ventricular activations. With the elimination of the accessory pathway, the delta wave disappeared and there was also the widening of the PR interval and normalization of the QRS complex.

ECGs during the follow up were repeatedly normal. At 9 years old, the patient began to present multiple episodes of paroxysmal tachycardia. The ECG showed at that time sinus rhythm with a ventricular pre-excitation probably due to the presence of a left posteroseptal AV accessory pathway (delta wave was negative in inferior leads and positive in lead V1). An electrophysiological study (EP study) was performed two years later when parents accepted the procedure. Through a right femoral venous access, a tetrapolar catheter (5 F) was placed at the right atrium. Continuous atrial stimulation with shorter length cycles or using two atrial extra stimuli, did not modify the pre-excitation pattern but induced a supraventricular tachycardia due to orthodromic AV reentrant tachycardia using retrogradely the accessory pathway (Figure 1).

Using a right femoral arterial access, an 8 F deflectable irrigated ablation catheter was inserted retroaortically to map the left AV ring. Mapping around mitral valve found shorter AV intervals during sinus rhythm and pre-excitation at the posteroseptal aspect of the mitral ring. However, no continuous AV signals could be recorded. Radiofrequency pulses (43°, 35 W) did not eliminate the pre-excitation. Using a second right femoral venous access, the ablation catheter was positioned in the posteroseptal aspect of the AV ring through the coronary sinus. Mapping of the mitral annulus from the coronary sinus was difficult because of the suspicion of a very dilated coronary sinus. Administration of contrast showed a very dilated coronary sinus. During sinus rhythm with pre-excitation, mapping deep inside the coronary sinus finally recorded the earliest continuous AV signal indicating the AP at posteroseptal localization. One single radiofrequency pulse (43°, 35 W) permanently eliminated the pre-excitation. Thirty minutes after the ablation, no recurrence of anterograde conduction was observed during atrial programmed electrical stimulation. Ventricular stimulation showed decremental VA conduction (Figure 1). No tachycardias were induced. At the 6 months follow up, the patient remains asymptomatic and without pre-excitation at control ECG.

## 3. Discussion

Persistent left superior vena cava (PLSVC) is the most frequent systemic venous anomaly, with a prevalence of nearly 0.21–0.6% in the general population [1,2]. It occurs during embryological development due to the lack of involution of the caudal portion of the left anterior cardinal vein and the left common cardinal vein, leading to a more venous return by the coronary sinus and widening of its structure [1,2]. Associated with congenital heart defects, the prevalence of PLSVC is 1.3–3.8%, seven times more than the general population [2]. It has been seen in cases of double outlet right ventricle, aortic coarctation, ventricular septal defects, atrial septal defects, atrioventricular septal defects, Fallot, partial anomalous pulmonary venous return, but uncommon in D-transposition of great arteries. PLSVC, along with other anomalies of CS (coronary sinus diverticula, aneurysm of CS), can be diagnosed by echocardiography, CS angiography, CT and cardiac MRI [4].

Nearly 0.3% of adults with supraventricular arrhythmias (SVT) in electrophysiological studies (EPs) have PLSVC. In fact, PLSVC can be present in all types of SVT (AVRNT, AVRT using accessory pathways, atrial tachycardia, atrial fibrillation and atrial flutter). The presence of PLSVC does not alter the distribution of frequency of the different types of SVT [5].

Although a PLSVC has no hemodynamical implications, it is important to recognize it, especially in electrophysiology, for several reasons. First, the CS anatomy and its spatial relations are important for guiding cardiac ablation [6]. The dilated CS alters the cardiac anatomy and the relation between Koch’s triangle and CS [7]. There is a displacement of the slow nodal pathway (SP) and the bundle of His that may complicate the ablation of the SP and increase the risk of AV block [8]. Additionally, the location of the SP could be found, even in 47.4% inside the CS. Secondly, in a dilated CS, there is an excessive movement of the ablation catheter inside the CS that makes mapping very difficult, especially if an accessory pathway is suspected [9]. Most of the APs found in relation with PLSVC are left pathways (posteroseptal, posterolateral, lateral) although right pathways can also be found (anteroseptal, midseptal) [4]. Some left accessory pathways will need ablation from inside the CS while others (such as the left lateral) are far from the dilated CS. It has been reported that there is more recurrence of AP after ablation in patients with PLSVC compared to normal hearts (17.6% vs. 4.1%, *p* = 0.047), but no difference in complications have been documented [2]. Thirdly, PLSVC has also been linked to the focus of atrial premature beats, focal atrial tachycardia or complex fragmented potentials associated with atrial fibrillation [2]. Fourth, although APs found in relation with PLSVC can be right pathways (anteroseptal, midseptal), most of these APs are left sided (posteroseptal, posterior, posterolateral, lateral) [4] and some of them will only be ablated from inside the dilated CS.

The present case is important as it shows a pediatric patient with PLSVC and a D-transposition of great arteries, which is an uncommon relation. The giant CS altered the spatial relations of the cardiac structures in the patient and avoided ablation of the AP from left heart access. After failure of ablation from retroaortic access, mapping and ablation from inside the coronary sinus was the solution to cure this patient. It shows the importance of knowing the impact of a PLSVC and the dilated CS for electrophysiology procedures to plan the mapping techniques in cases of left APs.

## 4. Conclusions

PLSVC affects EPs as it alters the CS anatomy, its relation to other structures of the conduction system and affects the stability of the catheter inside the dilated CS. In cases of left accessory pathways with complex mapping and/or ablation, working inside the dilated CS could be the solution.
